# India's adaptation of HIV services to ensure continuity of service delivery during the COVID‐19 pandemic

**DOI:** 10.1002/jia2.25964

**Published:** 2022-07-25

**Authors:** Anna Prasanna Kumari, M. R. Parthasarathy, Manish Bamrotiya, Ajay Reddy Enugu, Subash Chandra Ghosh, Chinmoyee Das, Nidhi Kesarwani, Alok Saxena

**Affiliations:** ^1^ Telangana State AIDS Control Society Hyderabad India; ^2^ Johns Hopkins University School of Medicine Baltimore Maryland USA; ^3^ YR Gaitonde Centre for AIDS Research and Education Chennai India; ^4^ Ministry of Health and Family Welfare National AIDS Control Organisation New Delhi India

1

The onset of the COVID‐19 pandemic and associated control measures have highlighted the dramatic impact that interventions implemented to control one pandemic could have on the management of other communicable and non‐communicable diseases [1]. While COVID‐19 pandemic‐associated mortality was devastating in India, particularly during the Delta surge, COVID‐19 also interrupted the delivery of HIV prevention and care programmes in India, forcing the government to adapt to ensure continuity.

India with an estimated 2.4 million people living with HIV (PLHIV) is home to the third largest population of PLHIV globally [2]. India has a general population HIV prevalence of 0.2%, with HIV concentrated among key populations. India is also home to the second largest number of reported COVID‐19 cases globally, with recent evidence suggesting that mortality due to COVID‐19 is perhaps six to seven times higher than reported, making India the country with the highest excess mortality due to COVID‐19 [3].

India delivers antiretroviral therapy (ART) free‐of‐charge to all PLHIV via public sector ART centres [4]. There are approximately 620 of these centres that provide treatment services to approximately 1.5 million PLHIV—these centres provide comprehensive HIV services, including clinical care, ART initiation, ART refills and viral load monitoring. Traditionally, PLHIV register for ART and receive services for their HIV care from a parent ART centre; to receive services such as ART refills at another ART centre, they would need to go through administrative processes to transfer out of their home centre and into a new centre. At each of these centres, ART is usually provided monthly except in the case of stable patients who qualify for multi‐month dispensation (MMD). MMD essentially means that 2–3 months of ART are dispensed at a given time. Stable patients may also receive their ART refills from over 1264 “link ART centres” (LACs). These LACs, which are linked to a parent (“nodal”) ART centre, were established to overcome logistical impediments to medication access, such as distance. The LACs do not initiate new patients on ART but provide adherence counselling, refills and monitor for adverse events, referring clients to the nodal ART centre at least once every 6 months for viral load monitoring, management of serious opportunistic infections and other services. This was the standard practice of ART delivery and viral load monitoring prior to the COVID‐19 pandemic.

In response to the emergence of COVID‐19 in March 2020, the Government of India announced a nationwide lockdown with less than half a day's notice. This required the HIV programme to rapidly adapt to ensure continuity of services. In response to these mobility restrictions, India's National AIDS Control Organisation (NACO) made major adaptations to ensure continuity of ART services, to the extent possible. First, they allowed community‐based, as well as home‐based, delivery of ART. Second, while MMD was exclusively for stable patients in the pre‐pandemic period, NACO revised their guidelines to ensure MMD was made available to all clients during the pandemic to limit the need for travel to ART centres and face‐to‐face contact with programme staff. Third, the nationwide lockdown resulted in mass migration from the larger cities to smaller towns and villages, which were oftentimes in a different state. As a result, the National AIDS Control Program revised their guidelines to allow ART refills from any ART centre, including certain private sector venues. We present below our experiences and lessons learned adapting HIV service delivery amidst the COVID‐19 pandemic in the south Indian state of Telangana. Telangana is home to about 158,000 PLHIV and the district of Hyderabad accounts for the most number of PLHIV in the state [5].

ACCELERATE, a PEPFAR/USAID‐funded programme, operates in five high‐burden districts of Telangana, provides technical assistance to NACO and assists with service delivery in the state of Telangana. Cumulatively, the five‐high‐burden districts in Telangana provide ART to over 50,000 PLHIV. Between April and October 2020, when India was in its strictest lockdown, the team performed almost 17,600 home deliveries (Figure 1a). Lists of clients on ART were provided to the team from the State AIDS Control Societies, and the programme team contacted clients to seek permission for home‐delivery—the most common reason for inability to contact clients was incorrect contact information. Among clients who consented to home delivery, outreach workers on two‐wheel vehicles delivered ART at the client's home or a place of the client's choosing. Common reasons for opting out of home delivery centred around confidentiality [6]. Specifically, it was often the case that family members and/or neighbours were unaware of their status. Further, while the majority of PLHIV welcomed the MMD option, it is noteworthy that a few preferred monthly dispensations, as they lived in hostels or shared living space with others who were unaware of their status, and cited difficulties in hiding multiple boxes of medications [6]. While these adaptations allowed us to ensure continuity of ART, we faced challenges with ensuring timely viral load monitoring. To complete a viral load test, clients had to physically visit an ART centre, which was challenging during periods of travel restrictions, thereby hampering viral load testing activities (Figure 1b). Similar to viral load testing, overall HIV diagnostic testing also declined, leading to reductions in numbers of new clients initiating ART.

**Figure 1:**
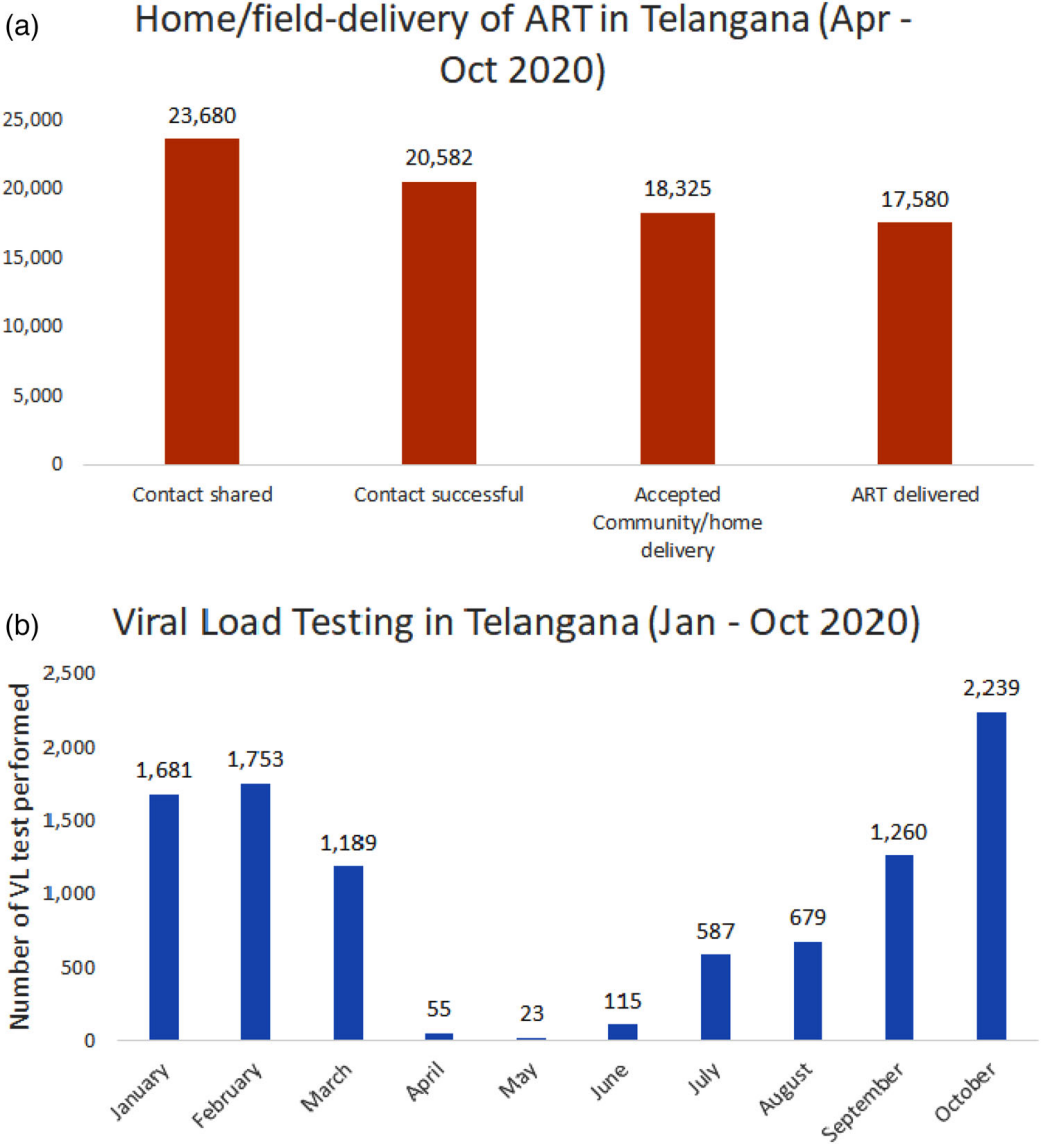
Field/home‐based delivery and viral load testing in five high prevalence districts of Telangana, India. Abbreviations: ART, antiretroviral therapy; VL, viral load.

Overall, these experiences offer valuable lessons for maintaining the continuity of programmes during and beyond a crisis. Most importantly, given the largely favourable response to home/field‐based delivery of ART as well as MMD for all clients, it might be worth considering home/field‐based ART delivery to clients as a standard of care to improve retention. One strategy that has been discussed is the use of community PLHIV networks coupled with tele‐counselling services to optimize adherence and treatment outcomes. Additionally, allowing clients to refill ART from multiple centres could assist in ensuring optimal adherence even outside of emergency public health situations, but needs to be backed with optimal documentation of refills dispensed. The rollout of SOCH (Strengthening Overall Care for HIV beneficiaries), a fully electronic platform to track clients from diagnosis to viral suppression, and planned integration of a unique identifier within this platform will allow for real‐time tracking of clients across the centres [7].

However, while India adapted their services to ensure continuity of ART delivery, there were other challenges that were more difficult to overcome, including access to HIV testing and viral load testing. To address these gaps, there are additional approaches that could be evaluated. First, the collection of dried blood spots at the time of field/community‐based ART dispensation could serve as a mechanism to quantify HIV viral load when specimen collection at ART centres themselves is challenging [8]. There are efforts underway to incorporate Dried Blood Spot‐based (DBS) HIV RNA quantification under the National Program. Second, the provision of HIV self‐test kits with access to tele‐counselling services for assisted HIV self‐testing could be an approach to ensure that PLHIV are diagnosed as early as possible. This could be followed by field/home‐based HIV confirmatory testing and initiation of ART, as has been demonstrated in trials of same‐day ART, with minimal adverse events [9]. The utility of self‐testing to deliver HIV screening services with linkage to confirmatory testing via tele‐counselling delivered by outreach workers is currently being implemented.

At this uncertain time in public health, it is critical that programmes share their best practices to facilitate learning across countries or regions to ensure that we do not let one new pandemic derail all the progress that has been made over the past four decades to combat HIV. COVID‐19 is not the first and will not be the last pandemic or crisis that we face. The lessons that we have learned should not be forgotten, but instead be carried forward to improve programmes generally and ensure that we are better prepared for the next challenge we face.

## COMPETING INTERESTS

The authors have no competing interests to declare.

## AUTHORS’ CONTRIBUTIONS

MRP and MB were responsible for the drafting of the manuscript with critical inputs from APK, CD, NK and AS. ARE oversaw all data collection and data processes. SCG oversaw the entire home delivery process. All authors provided inputs and approved the final version of the manuscript.

## FUNDING

We would like to acknowledge PEPFAR/USAID: 72038619CA00001 for support with the home‐delivery in Telangana.
